# Psychological Symptoms in Family Members of Brain Death Patients in Intensive Care Unit in Kerman, Iran

**DOI:** 10.5539/gjhs.v6n2p203

**Published:** 2014-02-10

**Authors:** Hakimeh Hosseinrezaei, Motahareh Pilevarzadeh, Masoud Amiri, Hossein Rafiei, Sedigheh Taghati, Mosadegheh Naderi, Mohammad Moradalizadeh, Milad Askarpoor

**Affiliations:** 1 Department of Medical and Surgical Nursing, Razi School of Nursing and Midwifery, Kerman University of Medical Science, Kerman, Iran; 2 Department of Medical and Surgical Nursing, School of Nursing and Midwifery, Jiroft University of Medical Science, Jiroft, Iran; 3 Social Health Determinants Research Center and Department of Epidemiology and Biostatistics, School of Health, Shahrekord University of Medical Sciences, Shahrekord, Iran; 4 Department of Intensive and Critical Care Nursing, School of Nursing and Midwifery, Shahrekord University of Medical Sciences, Shahrekord, Iran

**Keywords:** brain death, ICU, relatives/family members, anxiety, depression, stress, DASS- 42

## Abstract

**Aim:**

Having patients in Intensive Care Unit (ICU) remains an extremely stressful live event for family members, especially for those having to confront with brain death patients. The aim of present study was to determine the prevalence of depression, anxiety and stress among relatives of brain dead patients in ICU in Kerman, Iran.

**Methods:**

In a cross-sectional study, using DASS- 42 questionnaire, the symptoms of depression, anxiety and stress of family members of brain death patients were explored in Kerman, Iran.

**Results:**

Of 244 eligible family members, 224 participated in this study (response rate of 91%). Generally, 76.8%, 75% and 70.1% of family members reported some levels of anxiety, depression and stress, respectively. More specifically, the rate of severe levels of anxiety, depression and stress among the participants were 48.7%, 33%, and 20.1% respectively.

**Conclusion:**

Prevalence of depression, anxiety and stress in family members of brain death patients in ICU remains high. Health care team members, especially nurses, should be aware and could consider this issue in the caring of family members of brain death patients.

## 1. Introduction

An Intensive Care Unit (ICU) is a special area of care in each hospital which is mainly dedicated to the management of patients with life-threatening illnesses, injuries or complications (Hall, 2005). Having a patient in ICU is an extremely difficult experience for a concerned one (Frid et al., 2007). This is especially so for close relatives causing psychological symptoms such as feelings of anger, emotional pain, disbelief, guilt, suffering (mental), stress, anxiety, and depression (Azoulay et al., 2005; Davidson, 2007; Davidson et al., 2007; Frid et al., 2001; Frid et al., 2007; McAdam & Puntillo, 2009; Olsen et al., 2009; Paul & Rattray, 2008; Pillai et al., 2010)

In 2003, Azulay et al. (2005) surveyed factors that may cause symptoms of post-traumatic stress disorders in family members of ICU patients. They reported that 33.1% family members of critically ill patients had moderate to high risk for post-traumatic stress disorder. They also found that the rate of post-traumatic stress disorder is higher among relatives who felt they did not receive enough information in the ICU, who involved in end of- life decision-making, whose relative died in the ICU and after end-of-life decisions. In another study, Askari et al. (2013) compared psychological reactions of family members of patients in ICU and Coronary Care Unit (CCU) using the Depression, Anxiety and Stress Scale 42(DASS 42). They reported that 68%, 57.3% and 46.7% of the patients’ relatives had experienced anxiety, depression and stress, respectively. They also mention that family members of ICU patients experienced more levels of anxiety, depression and stress compared to family members of CCU patients. In a multicenter study, Puchrad et al. (2005) found that more than two-thirds of family members of critically ill patients in ICU experienced symptoms of anxiety or depression. In a randomized controlled trial, Jones et al. (2004) examined the effectiveness of a rehabilitation program on post-traumatic stress disorder rate in close relatives of ICU patients in United Kingdom. Their program comprised 6-week self-help manual containing information about recovery from ICU, psychological information and practical advice. They showed that rehabilitation program could not decrease the incidence of depression and anxiety among relatives of ICU patients.

Brain/cerebral death is also controversial ethical issue in ICU (Liao & Ito, 2010) and is defined as the irreversible loss of all functions of the brain, including the brainstem. The three essential findings in brain death are deep unresponsive coma, absence of brainstem reflexes, and absent respiratory effort confirmed by the apnea test (Goila & Pawar, 2009). Symptoms of depression, anxiety and stress in family members of brain death patients may be different from relatives of other critically ill patients. They usually encountered with organ donation that is very stressful event (Pelletier, 1992; Douglass & Daly, 1995; Evans, 1995). However, studies which specifically focused on the experiences of family members confronting brain death is very limited (Frid et al., 2007). To provide appropriate care for the family members of these patients, it is necessary for health care team members to assess primarily of the symptoms of relatives of patients (McAdam & Puntillo, 2009). This study thus conducted to fulfill this goal.

## 2. Methods

To address the above mentioned goal a cross-sectional study was conducted at Shahid Bahonar Center, a 302-bed teaching hospital affiliated with the Kerman University of Medical Science (KUMS) in southeast of Iran. The hospital has 39 ICU beds in three distinct ICUs (medical, surgical and trauma). Ethical approval was obtained from the research ethical centre of the KUMS (ethic code: K/90/526). Since the visiting hour in Shahid Bahonar ICUs is restricted, family members were eligible for the study if they came to the ICU to visit a patient with duration of staying more than 48 hours, aged 18 years and older, with no previous history of psychiatric disorder and psychotropic drug use (with or without physician prescription).

For each patient with brain death, we included the family member who was the most important person in decision making (spouse; parents/children; others). Before obtaining the participants’ consent, the goals of research and emphasis on confidentiality of data was comprehensively described to each participant. Between day 3 and day 5, each family representative was asked to complete a questionnaire.

Data was collected between January and September 2013, using DASS- 42 questionnaire. The DASS was developed by Lovibond and Lovibond and is a 42-item self report instrument designed to measure the three related negative emotional states of depression, anxiety and stress (Lovibond & Lovibond., 1995). Each of the three DASS scales contains 14 items, divided into subscales of 2-5 items with similar content. The *depression scale* examines dysphoria, hopelessness, devaluation of life, self-deprecation, lack of interest/involvement, anhedonia, and inertia. The *anxiety scale* examined autonomic arousal, skeletal muscle effects, situational anxiety, and subjective experience of anxious affect. The *stress scale* is sensitive to levels of chronic non-specific arousal which assesses difficulty relaxing, nervous arousal, and being easily upset/agitated, irritable/over-reactive and impatient. Subjects were asked to use 4-point severity/frequency scales to rate the extent to which they have experienced each state. Scores for depression, anxiety and stress are calculated by summing the scores for the relevant items (Askari et al., 2013; Afzali et al., 2007; DASS site, 2013; Lovibond & Lovibond., 1995). Validity and reliability of the Persian version of DASS- 42 was assessed in a previous study by Askari et al. (2013) and Afzali et al. (2007). Table 1 shows the scoring of DASS-42 in details.

**Table 1:** Scoring of DASS-42

	Normal	Mild	Moderat	Severe	Very Severe
**Depression**	0-9	10-13	14-20	21-27	28-42
**Anxiety**	0-7	8-9	10-14	15-19	20-42
**Stress**	0-14	15-18	19-25	26-33	34-42

## 3. Results

Family members of 101 patients participated in the current study. The mean age of patients was 44.8±21.5 years. Of the 244 eligible family members, 224 participated in this study (response rate of 91%). Of these, 45.1% were male and 54.9% were female. The mean age of participants was 35.6±13.3 years. Table 2 shows more demographic information about the relatives of patients. Generally, 76.8%, 75% and 70.1% of family members reported some levels of anxiety, depression and stress, respectively. The rate of severe levels of anxiety, depression and stress were 48.7%, 33%, 20.1%, respectively among relatives of these patients. Table 3 shows the score of anxiety, depression and stress in details. With increasing the age of family members of patients, the level of anxiety decreased but depression and stress were not affected by family members’ age (P<0.04). Level of participant anxiety, depression and stress between men and women were similar (p>0.05). Between family members, children reported higher levels of anxiety, depression and stress in comparison with fathers, mothers, sisters and brothers. This difference were statistically significant (P<0.03). The level of education of participants did not affect their level of anxiety, depression and stress (p>0.05).

**Table 2:** Participants demographic characteristic

Demographic characteristics’		Number and present	P value
**Gender**	Men	101(82.1%)	0.05
	Women	123 (17.9%)	
**Education**	Primary school	59 (26.3%)	0.05
	High school	99 (44.2%)	
	Graduate	59 (26.3%)	
	Post graduate	7 (3.1%)	
**Relationship to patient**	Father	18(8%)	0.04
	Mother	25(11.2%)	
	Sister	45(20.1%)	
	Brother	24(10.7%)	
	Child	77(34.4%)	
	Spouse	35 (15.6)	
**Request for organ donation**	Yes	216(14.5%)	0.05
	No	8 (14.5%)	

**Table 3:** Depression, anxiety, and stress levels in family members

	Depression		Anexity		Stress	
	Number	Percent	Number	Percent	Number	Percent
**Without symptom**	56	25	52	23.2	67	29.9
**Mild**	11	4.9	11	4.9	21	9.4
**Moderate**	32	14.3	29	12.9	39	17.4
**Severe**	51	22.8	23	10.3	52	23.2
**Very severe**	74	33	109	48.7	45	20.1

## 4. Discussion

To assess the symptoms of depression, anxiety and stress in family members of brain death patients, a group of critically ill patients with high rates of mortality was conducted. Our findings showed that the prevalence of depression, anxiety and stress among family members of brain death patients was consistently high.

Admission in ICU could be a stressful event for both patients and their family members. Having a patient with brain death in ICU could be more stressful for family members, because their patients are at higher risk of dying. Similar to our findings, Joes et al. (2004) in UK reported that close relatives of ICU patients had experienced higher levels of anxiety and depression. In their study, 62% and 31% of close relatives showed that the symptoms of anxiety and depression were lower compared to participants in this study. In our study, 76.8% and 75% of family members reported symptoms of anxiety and depression. This difference could be due to the difference in participants of two studies (patients of family members in our study had higher risk of dying in comparison with the patients of family members in Joes’ study) or different types of instruments used in two studies. For assessing anxiety and depression, we used DASS-42 questionnaire; however, Joes et al. (2004) had used hospital anxiety and depression scale. In the study of Askari in 2013, the symptoms of anxiety, depression and stress in ICU and CCU family members were studied. Similar to our findings, they reported that the rate of psychological symptoms was increased among the relatives of patients in critical care units. Askari et al. (2013) also found that the length of staying in ICU, sex, age and marital status may affect the psychological symptoms of family members. In another study, Pochard et al. (2005) explored the symptoms of anxiety and depression in relatives of critically ill patients in France. They reported that more than two-thirds of family members of critically ill patients who admitted to ICU, may suffer from symptoms of anxiety or depression which is similar to our findings. They also determined that three factors which may associate with the symptoms of anxiety and depression in family members includes; 1) patient-related factor (severity of patients illness and patient’s age and patient’s death); 2) family-related factor (the spouse); and 3) ICU-related factor (a room with more than one bed) Pochard et al., 2005). In the study of Bandari et al. (2013), the prevalence of anxiety among family members of ICU patients with use of State-Trait Anxiety Inventory (STAI) was assessed. Similar to our findings, they mentioned that the experience of anxiety may increase with having patients in ICU.

The policy of restriction in visiting of patients hospitalized in ICU may affect negatively on family members. Similar to most ICUs in Iran, ICUs that surveyed in present study used restricted policy for visiting of patients. Compared to restricted visiting, open visiting hours allows families to have maximum contact with their patient and may promote an atmosphere of openness and transparency (Hall, 2005). Garrouste-Orgeas et al. in 2008 determined the prevalence of symptoms of anxiety and depression in family members of patients who admitted to an ICU with open visiting policy. The prevalence of anxiety and depression reported by relatives were 49% and 29.5%, respectively.

In Iranian ICUs, a visit by the relatives usually is arranged by the critical care nurses, who caring patients. In fact, critical care nurses have an important role in programming for visiting in ICUs. In this regards, Simon et al. (1997) conducted a study on the nurses’ perceptions about open and restricted visiting policy and its effects on the health of patients, the patient’s family members, and the nurse in ICU. Nurses in Simon’s study perceived that restricted visiting hour may decrease the noise (83%) and may promote patients’ rest (85%) in ICU. They also perceived that open visiting hour beneficially affect the patients (67%), patient’s family members (88%) and may decrease family members anxiety symptom (64%). Berti et al. (2007) studied on the beliefs and attitudes of ICU nurses toward open visiting policy (Berti et al., 2007). They reported that most nurses did not want to liberalize the visiting policy of their ICU. They also mentioned that open visiting hour may tend to more time spending with patient’s family and giving more information to them (82.3%).

## 5. Conclusion

In this study, we found that prevalence of depression, anxiety and stress in family members of brain death patients in ICU is high. Some psychological symptoms may be improved with better communication between the health care team members and family members and the provision of more information regarding the patient (Day et al., 2013). Future research is recommended to study effect of visiting policy on relatives’ level of depression, anxiety and stress. Also studies need to examine psychological symptoms, 3, 6 and 12 month after diagnosis of brain death in family members of ICU patients.

## Limitation

One limitation of this study is convenience sampling. Furthermore, use of the self reported questionnaires may have led to an overestimation of some of the findings because of the variance that is common in different methods.

## References

[ref1] Afzali A, Delavar A, Borjali A, Mirzamani M (2007). Psychometric properties of DASS-42 as assessed in a sample of Kermanshah high school student. Journal of Research in Behavioural Sciences.

[ref2] Askari H, Forozi M, Navidian A, Haghdost A (2013). Psychological reactions of family members of patients in critical care unitsinZahedan. J Research Health.

[ref3] Azoulay E, Pochard F, Kentish-Barnes N, Chevret S, Aboab J, Adrie C, Schlemmer B, FAMIREA Study Group (2005). Risk of post-traumatic stress symptoms in family members of intensive care unit patients. Am J Respir Crit Care Med.

[ref4] Bandari R, Heravi-Karimooi M, Rejeh N, Zayeri F, Mirmohammadkhani M, Montazeri A (2013). Anxiety prevalence and its associated demographic factors in family members of patients hospitalized in the intensive care unit: a cross-sectional study in Tehran. Daneshvar Medicine.

[ref5] Berti D, Ferdinande P, Moons P (2007). Beliefs and attitudes of intensive care nurses toward visits and open visiting policy. Intensive Care Med.

[ref6] Davidson J. E (2009). Family-centered care: meeting the needs of patients’ families and helping families adapt to critical illness. Crit Care Nurse.

[ref7] Davidson J. E, Powers K, Hedayat K. M, Tieszen M, Kon A. A, Shepard E, Armstrong D, American College of Critical Care Medicine Task Force 2004-2005, Society of Critical Care Medicine (2007). Clinical practice guidelines for support of the family in the patient-centered intensive care unit: American College of Critical Care Medicine Task Force 2004–2005. Crit Care Med.

[ref8] Day A, Haj-Bakri S, Lubchansky S, Mehta S (2013). Sleep, anxiety and fatigue in family members of patients admitted to the intensive care unit: a questionnaire study. Critical Care.

[ref9] Douglass G. E, Daly M (1995). Donor families experience of organ donation. Anaesthesia and Intensive Care.

[ref10] Evans D (1995). Brain death: the family in crisis. Intensive and Critical Care Nursing.

[ref11] Frid I, Bergbom-Engberg I, Haljamäe H (2001). No going back: narratives by close relatives of the brain dead patient. Intensive Crit Care Nurs.

[ref12] Frid I, Haljamäe H, Ohlén J, Bergbom I (2007). Brain death: close relatives’ use of imagery as a descriptor of experience. J Adv Nurs.

[ref13] Garrouste-Orgeas M, Philippart F, Timsit J. F, Diaw F, Willems V, Tabah A, Carlet J (2008). Perceptions of a 24-hour visiting policy in the intensive care unit. Crit Care Med.

[ref14] Goila A. K, Pawar M (2009). The diagnosis of brain death. Indian J Crit Care Med.

[ref15] Hall J. B, Schmidt A. G, Wood L. D (2005). Principal of Critical Care.

[ref16] Jones C, Skirrow P, Griffiths R. D, Humphris G, Ingleby S, Eddleston J, Gager M (2004). Post-traumatic stress disorder-related symptoms in relatives of patients following intensive care. Intensive Care Med.

[ref17] Liao S, Ito S (2010). Brain death: ethical challenges to palliative care concepts of family care. J Pain Symptom Manage.

[ref18] Lovibond P. F, Lovibond S. H (1995). The structure of negative emotional states: Comparison of the Depression Anxiety Stress Scales (DASS) with the beck depression and anxiety inventories. Behav Res Ther.

[ref19] Lovibond S. H, Lovibond P. F (1995). Manual for the Depression Anxiety Stress Scales.

[ref20] McAdam J. L, Puntillo K (2009). Symptoms experienced by family members of patients in intensive care unit. Am J Crit Care.

[ref21] Olsen K. D, Dysvik E, Hansen B. S (2009). The meaning of family members’ presence during intensive care stay: a qualitative study. Intensive Crit Care Nurs.

[ref22] Paul F, Rattray J (2008). Short- and long-term impact of critical illness on relatives: literature review. J Adv Nurs.

[ref23] Pelletier M. L (1992). The organ donor family members’ perception of stressful situations during the organ donation experience. Journal of Advanced Nursing.

[ref24] Pillai L, Aigalikar S, Vishwasrao S. M, Husainy S. M (2010). Can we predict intensive care relatives at risk for posttraumatic stress disorder?. Indian J Crit Care Med.

[ref25] Pochard F, Darmon M, Fassier T, Bollaert P. E, Cheval C, Coloigner M, Azoulay E, French FAMIREA study group (2005). Symptoms of anxiety and depression in family members of intensive care unit patients before discharge or death: a prospective multicenter study. J Crit Care.

[ref26] Simon S. K, Phillips K, Badalamenti S, Ohlert J, Krumberger J (1997). Current practices regarding visitation policies in critical care units. Am J Crit Care.

